# Graphene quantum dots promote migration and differentiation of periodontal ligament stem cells

**DOI:** 10.3389/fchem.2023.1213507

**Published:** 2023-11-13

**Authors:** Yan Liang, Wanshan Gao, Sicheng Deng, Dongyan Wu, Yiling Jiang, Yishan Zhang, Rongmin Qiu

**Affiliations:** Guangxi Key Laboratory of Oral and Maxillofacial Surgery Disease Treatment, Guangxi Health Commission Key Laboratory of Prevention and Treatment for Oral Infectious Diseases, Guangxi Key Laboratory of Oral and Maxillofacial Rehabilitation and Reconstruction, Guangxi Clinical Research Center for Craniofacial Deformity, College of Stomatology, Hospital of Stomatology, Guangxi Medical University, Nanning, China

**Keywords:** graphene quantum dots, nanomaterials, periodontal ligament stem cells, periodontitis, bone tissue engineering, osteogenic differentiation

## Abstract

Graphene and its derivatives have attracted much attention as nanomaterials in bone tissue engineering because of their remarkable ability to induce cell osteogenic differentiation. However, graphene quantum dots (GQDs), as graphene derivatives, little is known about their osteodifferentiation- and osteoinduction-promoting capabilities, especially in the restoration of bone defect caused by periodontitis. Therefore, there is a growing need to investigate the effect of GQDs on periodontal ligament stem cells (PDLSCs). Here, we postulated that GQDs are a promising biocompatible nanomaterial that facilitated the migration and differentiation of PDLSCs, and use laboratory methods like CCK-8, transwell experiments, qRT-PCR, Alizarin red staining and immunofluorescence staining to evaluate. Our experiments confirmed that GQDs did not inhibit cell viability, with most cells remaining viable even at GQDs concentrations of up to 30 μg mL^-1^. Moreover, GQDs were found to significantly enhance PDLSC migration, with the peak effect observed at concentrations of 5 and 10 μg mL^-1^. Furthermore, GQDs accelerated osteoblastic differentiation in PDLSCs and induced the mineralization of calcium nodules. Additionally, GQDs were shown to promote fibroblast differentiation in PDLSCs compared to the control group. Thus, GQDs not only possessed low cytotoxicity and good biocompatibility, but also displayed the beneficial capability to migration and differentiation of PDLSCs, which indicated GQDs might be a potential nanomaterial for bone regeneration.

## 1 Introduction

Periodontitis is a chronic, non-communicable disease with dental plaque biofilm as the main pathogenic factor (Lang and Bartold, 2018; Curtis et al., 2020). Presently, adequate therapeutic effects are rarely achieved for patients with severe periodontitis, and the costs of treatment and sequential care can be extremely high, resulting in a plausible negative impact on general health (Papapanou et al., 2018). Therefore, the focus of researches has evolved over time from a respective to a reparative or a regenerative approach (Caffesse and Echeverría, 2019; Tavelli et al., 2020). The reparative and regenerative approach are a part of tissue engineering. Currently, tissue engineering, especially bone tissue engineering, is considered a potential therapeutic method to regenerate and reconstruct periodontal tissue. There are three elements consisted in tissue engineering: stem cells, growth factors, and biomaterial scaffolds. Summarily, bone tissue engineering requires migration and recruitment of osteoprogenitor cells to remodel tissues under the guidance of growth factors and biomaterial scaffolds. (Bacakova et al., 2018).

Periodontal ligament (PDL) stem cells (PDLSCs) are an important cellular constituent of the PDL, contributing not only to PDL repair but also to the restoration of lost bone and cementum. A prominent feature of PDLSCs is the ability to secrete molecules that can regulate the extent of mineralization and prevent the fusion of tooth root with surrounding bone, making them a unique and first choice in tissue engineering research (Nanci and Bosshardt, 2006). The key objective in bone tissue engineering is to manipulate the function of growth factors and biomaterial scaffolds in a controlled manner to guide the satisfactory fate of PDLSCs.

Numerous materials have been investigated for their regenerative potential in treating periodontal defects, either in isolation or in various combinations. However, the majority of available bone grafts and substitute materials are primarily osteocompatible rather than osteoconductive. Moreover, many studies focusing on bone grafts encounter challenges related to ethical approval, limited availability of sources, and concerns about biological safety. Both alloplastic materials (such as tricalcium phosphate, hydroxyapatite, and bioglass) and biological factors (predominantly enamel matrix derivatives) have demonstrated limited effectiveness in periodontal regeneration (Sculean et al., 2015).

Graphene is a two-dimensional carbon nanomaterial with hexagonal honeycomb lattice in sp^2^ hybrid orbit (Novoselov et al., 2004). Graphene family materials possess exceptional and unique properties derived from their chemical structure, such as ultra-electronic flexibility, tremendous surface area, high mechanical strength, intrinsic mobility, and excellent thermal conductivity. Apart from the intrinsic mechanical properties of graphene, certain graphene family materials were found to be able to enhance stem cell proliferation and osteogenesis. It was (Nayak et al., 2011) proved that graphene and its derivatives are a promising biocompatible scaffold that accelerate the specific differentiation of human mesenchymal stem cells (MSCs) into bone cells even in the absence of classical growth factors, such as BMP-2 (Bone morphogenetic protein 2). Another study (Lee et al., 2015) constructed reduced graphene oxide-coated hydroxyapatite composites and confirmed the potential of graphene-based particulate materials to stimulate the spontaneous differentiation of MSCs via laboratory methods, such as detection of alkaline phosphatase (ALP) activity, mineralization of calcium and phosphate, and activity of osteogenic markers. In the study of the mechanism underlying graphene’s osteogenic ability, it was found that graphene and graphene oxide were a pre-concentrate platform for typical osteogenic inducers, such as dexamethasone and *β*-glycerolphosphate, which are ascribed to different degrees of π-π stacking and electrostatic and hydrogen bonding (Lee et al., 2011).

Graphene quantum dots (GQDs) are the latest addition to the graphene family. They are atomically thin in height and nanometers of size in lateral dimension, which make themselves be classified into nanomaterials. Various nanomaterials (including 0D, 2D, and 3D structures) are used as the filler material in scaffolds. The remarkable mechanical strength of graphene is advantageous when it is applied as a reinforcing filler in many tissue engineering materials. A hydroxyapatite hydrogel was incorporated with 2D graphene oxide for the mechanical and biological effects in bone tissue engineering. The resulting porous graphene-HA gels show electrical conductivity, biocompatibility, and augmented mechanical strength, making these soft scaffolds an excellent candidate for bone tissue engineering (Xie et al., 2015). As graphene derivatives, GQDs have the same osteogenic properties, these complex nanoparticles possess the ability to enhance osteogenic differentiation and regulation of the immune microenvironment (Wang et al., 2022). It was confirmed that GQDs not only exhibited a positive influence on the osteogenic differentiation of MSCs through BMP (Bone morphogenetic protein) and TGF-*β* (transforming growth factor-*β*) relative signaling pathways but also enabled MSCs to differentiate into adipocytes (Qiu et al., 2016). Furthermore, GQDs are advantaged in low toxicity and excellent biocompatibility (Tabish et al., 2018). Researchers hypothesized that the smaller size and high oxygen content of GQDs compared to GO or rGO might be the primary cause of low damage to cell viability during GQDs activity (Vahdat et al., 2019). Although the potential use of GQDs in modulating MSC differentiation is evident, the interactions between GQDs and PDLSCs—the major cell resource to form alveolar bone have not been studied in detail yet, and the interaction effect of PDLSCs cellular behavior and differentiation in the presence of GQDs nanoparticles remain largely unknown. Consequently, various parameters such as viability, migration, and differentiation potential of PDLSCs upon exposure to GQDs must be systematically evaluated.

In this study, we conducted experiments to investigate these concepts and concluded that GQDs expressed little toxicity in the concentration of 0–30 μg mL^-1^ and are relatively biocompatible. Further experiments indicated that GQDs enhanced the migration, osteogenic and fibroblast differentiation abilities of PDLSCs, thereby revealing their potential applications in nanomaterials for bone tissue engineering, especially in periodontics.

## 2 Materials and methods

### 2.1 Materials

The GQDs used in this experiment were solved in water, containing a little DMF. They were purchased from XFNANO Co., Ltd. (Nanjing, China). Transwell insert was purchased from Corning Inc. (Corning, NY, USA). Dulbecco’s Modified Eagle Medium (DMEM), fetal bovine serum (FBS) PBS, penicillin-streptomycin liquid and trypsin were obtained from Wisent Inc. (Quebec, Canada). We purchased 0.2% Alizarin Red S Staining Solution (ARS) from Beyotime (Shanghai, China). The molecular marker antibodies (CD34, CD90, CD146, and STRO-1) were obtained from BD Biosciences (San Jose, CA, USA) and Thermo Fisher Scientific (Waltham, MA, USA). Osteogenic and adipocyte differentiation medium was purchased from Cyagen Co., Ltd. (Suzhou, China). Cell counting kit-8 (CCK-8) was purchased from FUDE Biological Technology Co., Ltd. (Hangzhou, China). We obtained 4% polyformaldehyde, tween 20, triton X-100, anhydrous ethano and 0.1% crystal violet staining solution from Solarbio (Beijing, China). Trizol, chloroform, and isopropyl alcohol were obtained from Chuandong chemical reagent Co., Ltd. (Chongqing, China). We purchased DEPC water from Biosharp (Guangzhou, China). HiScript III RT SuperMix for quantitative polymerase chain reaction (qPCR) (+gDNA wiper) and ChamQ Universal SYBR qPCR Master Mix were purchased from Vazyme (Nanjing, China). Anti-Collagen Ⅰ antibody and goat anti-rabbit IgG H&L were purchased from Abcam (Cambridge, UK).

### 2.2 Characterization of GQDs

GQDs at a concentration of 1 mg mL^-1^ were sterilized through a 0.22 μm filter membrane. The GQDs composition was characterized using Fourier-transform infrared spectroscopy (FT-IR; Thermo Scientific Nicolet iS10). The GQD morphology was observed via transmission electron microscopy (Talos™ F200S, Thermo Fisher) at an acceleration voltage of 300 KV. Besides, UV-vis of GQDs was tested in UV-Visible Absorption Spectrometer (UV-2550, Shimadzu, Japan).

### 2.3 Isolation and characterization of PDLSCs

A modified method was used to isolate and characterize PDLSCs based on previous reference (Shi et al., 2005; Seo et al., 2016). PDL tissue was taken from healthy premolars of adolescents aged 12–18 years, whose premolars were clinically removed for orthodontic purposes. The premolars were immediately placed into a 4°C pre-cooled solution containing penicillin, streptomycin, and serum, after which they were taken to the laboratory. PDL tissue extraction was finished within 2 h, as recommended. Informed consent was obtained from the guardians before healthy premolars were removed.

PDL tissue was gently scraped from the middle third root surface with sterile blades, and placed onto configured medium (DMEM-F12 containing 20% FBS, 1% penicillin-streptomycin liquid). Culture medium was renewed every 3–5 days, as recommended. Primary cells were collected and cultured using the limiting dilution method to separate PDLSCs.

Before the study, it was approved by the Ethics Committee of Guangxi Medical University (NO. 20200050).

Flow cytometer was used to analyze the positive rate of molecular markers (CD34, CD90, CD146, and STRO-1) on the surface of stained cells. Identification of osteogenic differentiation ability was conducted as follows: PDLSCs were inoculated in a 6-well plate at a density of 2×10^4^ cells cm^-2^ and cultivated in complete medium. Osteogenic differentiation medium (Cyagen) replaced the complete medium when the cell confluence reached 60%–70%. PDLSCs were continued cultured for 2–4 weeks. After osteogenic induction, cells were fixed with 4% paraformaldehyde for 30 min, stained with ARS for 3–5 min, and then rinsed with PBS 2–3 times. Identification of adipogenic differentiation ability was conducted as follows: PDLSCs were inoculated in a 6-well plate at a density of 2×10^4^ cells cm^-2^, and cultivated in complete medium until the cell confluence reached 100% or was over-fused. The cells were then continually cultured in adipocyte differentiation medium. After induction, cells were fixed with 4% paraformaldehyde for 0.5 h, stained with Oil Red O for 30 min, and then rinsed with PBS 2–3 times.

### 2.4 Cell viability assay

In this study, the CCK-8 method was used to determine the viability of PDLSCs cultured in gradient GQDs concentration media (0, 5, 10, 15, 20, 25, and 30 μg mL^-1^) to reflect their toxicity. A third-generation PDLSCs (3000 cells well^-1^) were cultured in 96-well plates with complete medium for 24 h and then replaced it with GQDs medium at different concentrations (100 μL for each well), all in triplicate. 10 μL CCK-8 was added to each well and incubated for 3 h on day 0, 1, 3, 5, and 7, and the absorbance was measured at 450 nm using a microplate reader (TECAN Infinite M200Pro, canton of Zürich, Switzerland).

### 2.5 Cell migration assay

The Transwell experiment was used to detect the migration ability of PDLSCs with/without the influence of GQDs. The steps performed were as follows:

A third-generation PDLSCs were suspended in three concentrations: 5×10^4^ cells mL^-1^, 1×10^5^ cells mL^-1^, and 2×10^5^ cells mL^-1^. The above 3 cell concentrations in 200 μL serum-free media were added to the Transwell inserts (pore size, 8.0 μm). Further, 700 μL complete medium (DMEM-F12 medium containing 10 % FBS, 1% penicillin-streptomycin liquid) was added to a 24-well plate. Transwell inserts were placed in the 24-well plate. The cells in the inserts were fixed with 4% paraformaldehyde and stained with 0.1% crystal violet staining solution at 6, 12, and 24 h, respectively.

PDLSCs concentration was adjusted with serum-free medium to 2×10^5^ cells mL^-1^. Further, 100 μL cell suspension and 700 μL complete medium containing GQDs were separately added to the Transwell inserts and the lower chamber in the 24-well plate. The GQDs concentrations were set at 0, 5, 10, 15, 20, 25, and 30 μg mL^-1^, and the experiment was repeated thrice for each concentration. Cells were fixed with 4% paraformaldehyde and stained with 0.1% crystal violet staining solution after 24 h.

### 2.6 Cell differentiation assay

qRT-PCR was used to determine the effects of GQDs and osteogenic medium on PDLSCs differentiation, and the cells were divided into the following four groups: control group (normal complete medium), osteo group (osteogenic differentiation medium), 5GQDs group (normal complete medium with 5 μg mL^-1^ GQDs), and 5GQDs-osteo group (osteogenic differentiation medium with 5 μg mL^-1^ GQDs). The osteo group, 5GQDs group, and 5GQDs-osteo group were set as experimental groups, while cells cultured by normal complete medium were treated as control group.

After PDLSCs on the third generation were cultured in 6-well plates by above medium for 7, 14, and 21 days, respectively, total RNA was extracted, and RNA reverse transcription and qPCR were used to detect the relative expression of marker genes, including periodontal ligament relative fiber marker gene (Scleraxis), cementum marker gene (Cementum Protein 1, CEMP-1) and osteogenic differentiation marker genes (runt-related transcription factor 2, RUNX2; Osteocalcin, OCN; collagen type I, COL-I), GAPDH was chosen as the housekeeping gene for its high and constant expression in most tissues. The relevant gene sequences were found in the NCBI database and designed using the Oligo V 7.56 (Table 1). NCBI BLAST was used to identify the specificity of the primers.

**TABLE 1:** Primer sequences used in qPCR.

Name	Forward	Reverse	
GAPDH	TCT​CCT​CTG​ACT​TCA​ACA​GCG​ACA	CCC​TGT​TGC​TGT​AGC​CAA​ATT​CGT	
Scleraxis	GAG​AGG​TCC​ACA​CAG​CAC​AAG​AAC	GCG​GGC​ACA​GGC​GAG​TAT​TTA​G	
CEMP-1	AGC​TCT​GGG​TTT​TAG​CTG​AGG	GCC​GAT​GTG​TTA​GAG​GTT​GAG	
RUNX2	CCA​CTG​AAC​CAA​AAA​GAA​ATC​CC	GAA​AAC​AAC​ACA​TAG​CCA​AAC​GC	
ALP	AGC​TTC​AAA​CCG​AGA​TAC​AAG​CA	CTG​TTC​AGC​TCG​TAC​TGC​AT	
OCN	CCC​AGT​CCC​CTA​CCC​GGA​T	AGC​AGA​GCG​ACA​CCC​TAG​ACC	
COL-Ⅰ	GAG​GGC​AAC​AGC​AGG​TTC​ACT​TA	TCA​GCA​CCA​CCG​ATG​TCC​A	

qPCR, quantitative polymerase chain reaction.

Alizarin red staining (ARS) was used to detect the effect of GQDs on the formation of mineralized nodules in PDLSCs. The cells were divided into two groups and cultured: osteo group (cultured by osteogenic differentiation medium), and 5GQDs-osteo group (cultured by osteogenic differentiation medium with 5 μg mL^-1^ GQDs). At 7 and 14 days after introduction, cells were stained with ARS to observe mineralization results.

### 2.7 Immunofluorescence staining

For immunofluorescence staining, PDLSCs on the third generation were divided into the following four groups: control group (normal complete medium), osteo group (osteogenic differentiation medium), 5GQDs group (normal complete medium with 5 μg mL^-1^ GQDs), and 5GQDs-osteo group (osteogenic differentiation medium with 5 μg mL^-1^ GQDs). Multiple pores were set based on groups. PDLSCs were seeded in a 6-well plate and cultivated for 7, 14, and 21 days. The cells were preserved with 4% paraformaldehyde, incubated with 0.2% Triton X-100 for 10 min, and then incubated with PBST supplemented with 10% goat serum for 20 min. PDLSCs were treated with Anti-Collagen I antibody at 4°C overnight. After PBS washing, PDLSCs were treated with secondary antibody for 1 h, and cell nuclei were counterstained with DAPI. After washing with anti-fluorescence quenching agent, the green fluorescence results were visualized using a fluorescence microscope (Olympus FV3000, Nikon, Japan), and the pictures were recorded.

### 2.8 Statistical analysis

All experiments were repeated in triplicate. Statistical analysis was performed using SPSS 25.0 (IBM, USA), and the experimental data were presented as the mean ± standard deviation. One-way ANOVA and nonparametric test were used to compare the means between groups in experiments. All tests were two-sided, and *p* < 0.05 was considered statistically significant.

## 3 Results and discussion

### 3.1 Characterization of GQDs

**FIGURE 1:**
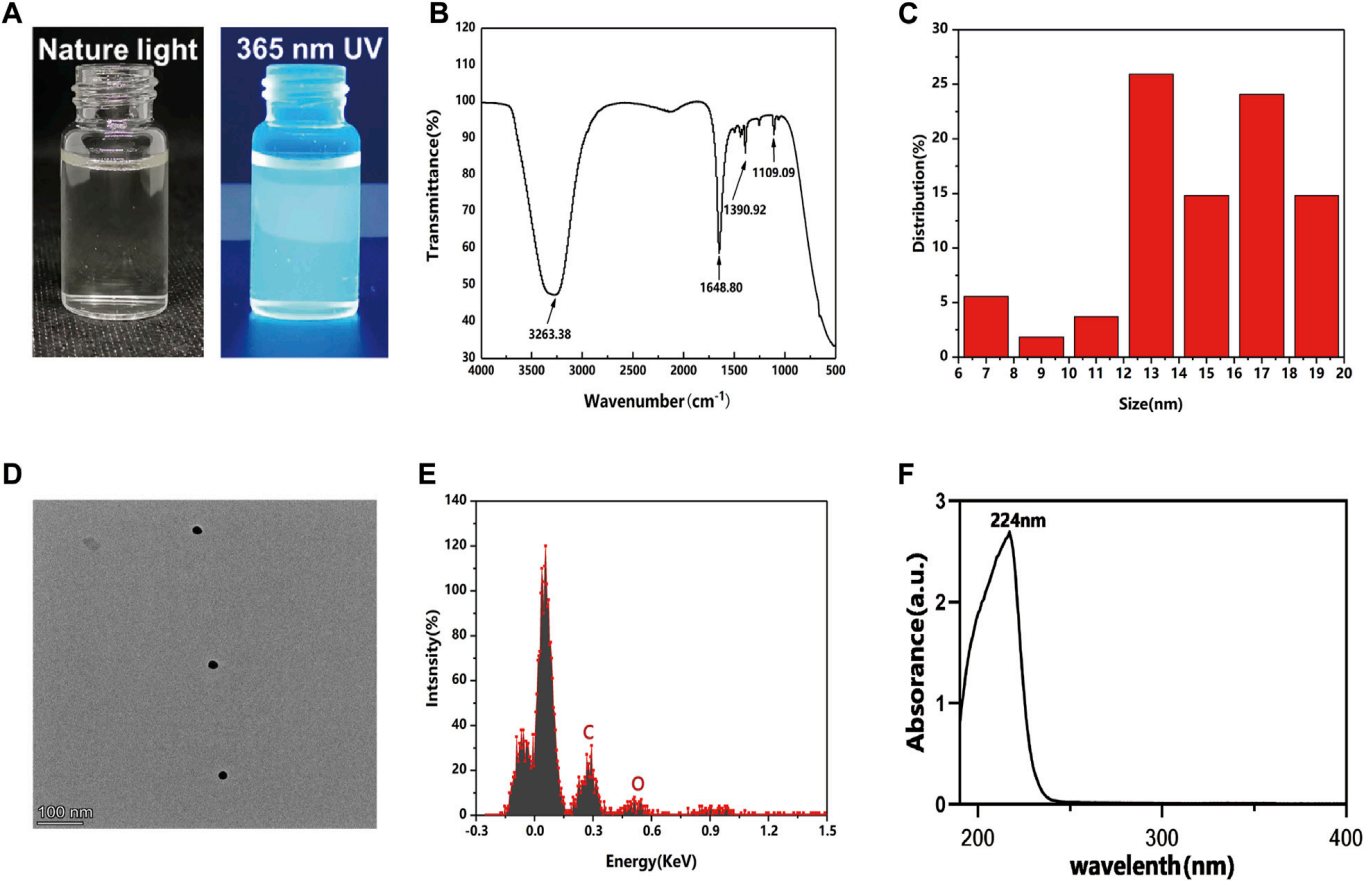
Characterization of GQDs. **(A)** Photos of GQDs aqueous solution under natural light (left), Green fluorescence could be observed from GQDs aqueous solution under UV light excitation at 365 nm (right). **(B)** Fourier transform infrared spectra of GQDs. **(C)** Size distribution of GQDs in water. **(D)** TEM images of GQDs. **(E)** EDS analysis of GQDs in water. **(F)** UV-vis absorption of GQDs.

The photographs of GQDs in aqueous solution under natural and 365 nm ultraviolet (UV) light are shown in Figure 1A. Green fluorescence could be observed with 365 nm UV light excitation. FT-IR results showed the position of the weak absorption peak at ∼1109 cm^-1^ and sharp absorption peak at ∼1390 cm^-1^, corresponding to the stretching vibrations of the C-O and C-H bonds; the peak at ∼1648 cm^-1^ was due to the C=C stretching vibrations, and the wide absorption peak at ∼3263 cm^-1^ indicated the presence of O-H and CO_2_H stretching (Figure 1B). The obtained FTIR results proved the evident features of carbon and oxygen relative functional groups similarly to previous studies (Wang et al., 2020; Thi et al., 2022). GQDs in the experiment showed a diameter size less than 20 nm (Figure 1C). Figure 1D shows the high-resolution transmission electron microscope (TEM) image of GQDs, which contained positive hexagonal carbon atoms and formed a honeycomb graphene-like lattice structure. Energy Dispersive Spectrometer (EDS) analysis showed that the element composition of GQDs. Carbon and oxygen were detected in GQDs samples. (Figure 1E). The UV-vis spectrum of GQDs (Figure 1F) shows a significant absorption peak at 224 nm (corresponding to the π→π* transition in the sp^2^ domain region). The characterization of GQDs revealed their basic physical and chemical properties, laying a foundation for further application in biomedicine (Mansuriya and Altintas, 2020; Zang et al., 2021; Lee et al., 2022).

### 3.2 Characterization of PDLSCs

**FIGURE 2:**
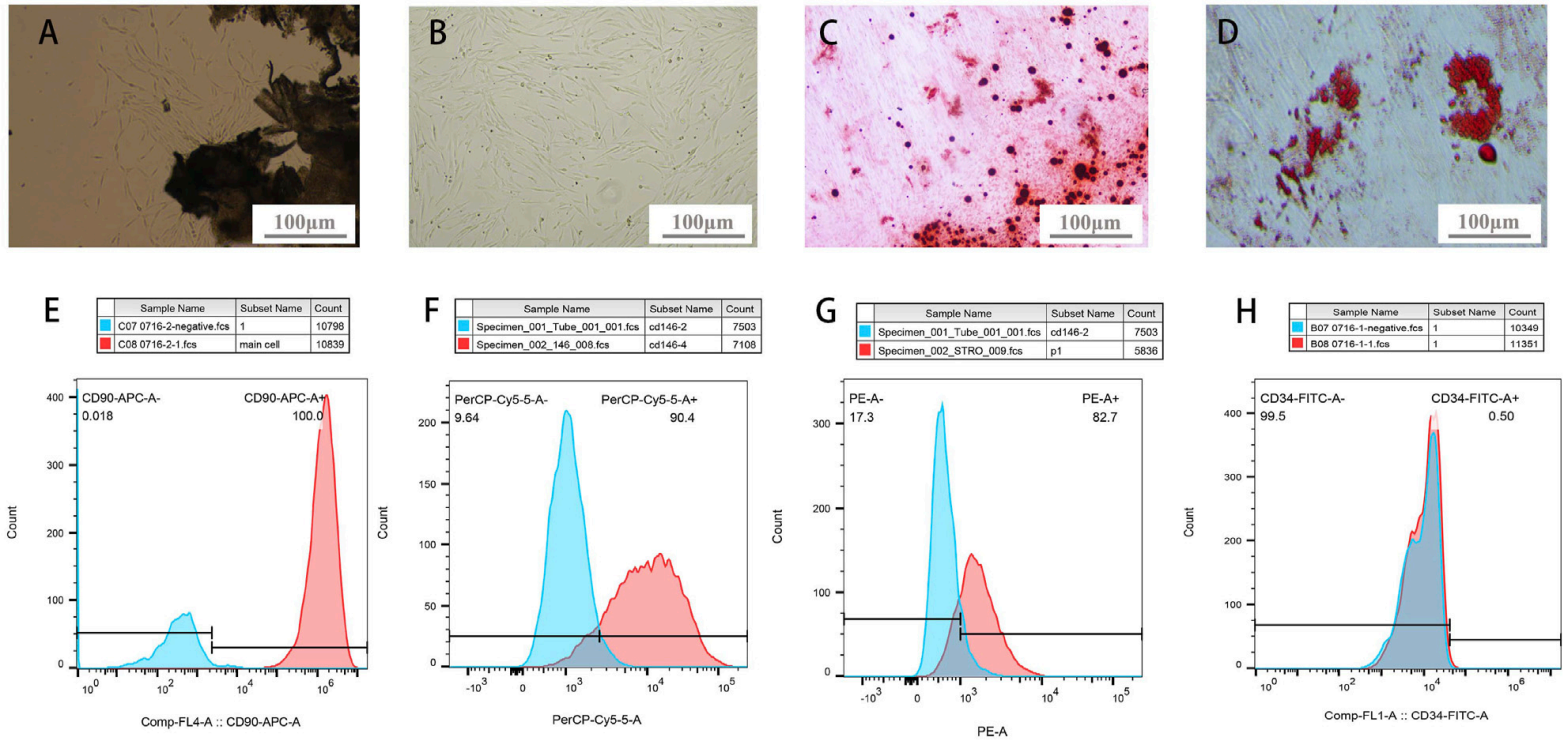
PDLSCs identification. **(A)** Primary culture of PDLSCs: cells crawled out from PDL tissue **(B)** Colony culture of PDLSCs: cells clusters stem from 1 cell **(C)** bright red- or dark red-staining calcified deposits formation after osteogenic induction of PDLSCs **(D)** adipocyte lipid droplets formation after adipogenic induction of PDLSCs. **(E–H)** Molecular markers expressions on the surface of PDLSCs (E-CD90, F-CD146, G-STRO-1, H-CD34).

PDLSCs were elongated and spindle-shaped morphology, with an enlarged middle region of the cell body (Figure 2A). PDLSCs were separated and purified in a 96-well plate using the limiting dilution method. Subsequently, monoclonal culturing was performed. The PDLSCs showed a uniform long spindle shape under the microscope (Figure 2B).

After osteogenic and adipogenic induction, PDLSCs were stained with ARS and Oil Red O. Cells secreted bright red- or dark red-staining calcified deposits (Figure 2C). Oil Red O staining showed that the cells secreted a large number of lipid droplets, which aggregate in a string of beads (Figure 2D). This demonstrated that the purified cells possess the osteogenic and adipogenic characteristics of stem cells. Under defined culture conditions (Seo et al., 2016).

Regarding the analysis of molecular markers on the surface of PDLSCs, the positive rates of CD90, CD146, STRO-1, and CD34 were 100%, 90.4%, 82.7%, and 0.5%, respectively (Figures 2F–I). CD90, CD146, and STRO-1 are widely recognized MSC surface-specific molecular markers. Isolated cells with a STRO-1 and CD146 markers has clonogenic properties and multi-lineage potentials, meanwhile, CD90 is a kind of stromal cells markers that expressed by PDLSCs (Ivanovski et al., 2006). And CD34 is a hematopoietic stem cell surface-specific molecular marker. The results demonstrated that cells from human PDL were mesenchymal stem cells, which had abundant differentiation potential (Bi, et al., 2007).

### 3.3 Cell viability of PDLSCs

**FIGURE 3:**
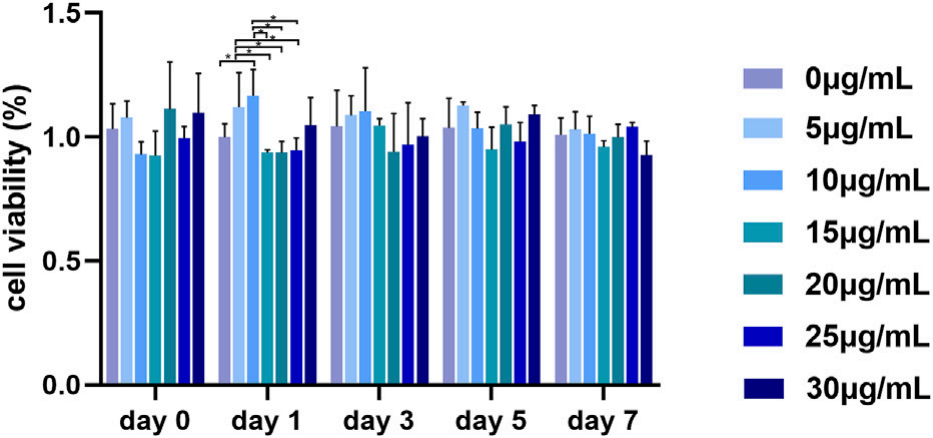
Viability of PDLSCs cultured by GQDs mediums. (*p < 0.05).

As shown in Figure 3, there was no obvious significant difference in the viability rate among the gradient GQDs concentration groups in the same day. The overall viability of PDLSCs did not decrease significantly in the concentration range of 0–30 μg mL^-1^, indicating that GQDs exhibited low cytotoxicity and excellent biocompatibility, which is consistent with previous research (Tabish et al., 2018; Vahdat et al., 2019; Zhang et al., 2019; Lee et al., 2020). Studies have also revealed that GQDs possess lower toxicity owing to their small size and high oxygen content, thus making them easier to metabolize *in vivo* and *in vitro* compared with other graphene family derivatives, such as GO and rGO (Vahdat et al., 2019).

### 3.4 Migration of PDLSCs

**FIGURE 4:**
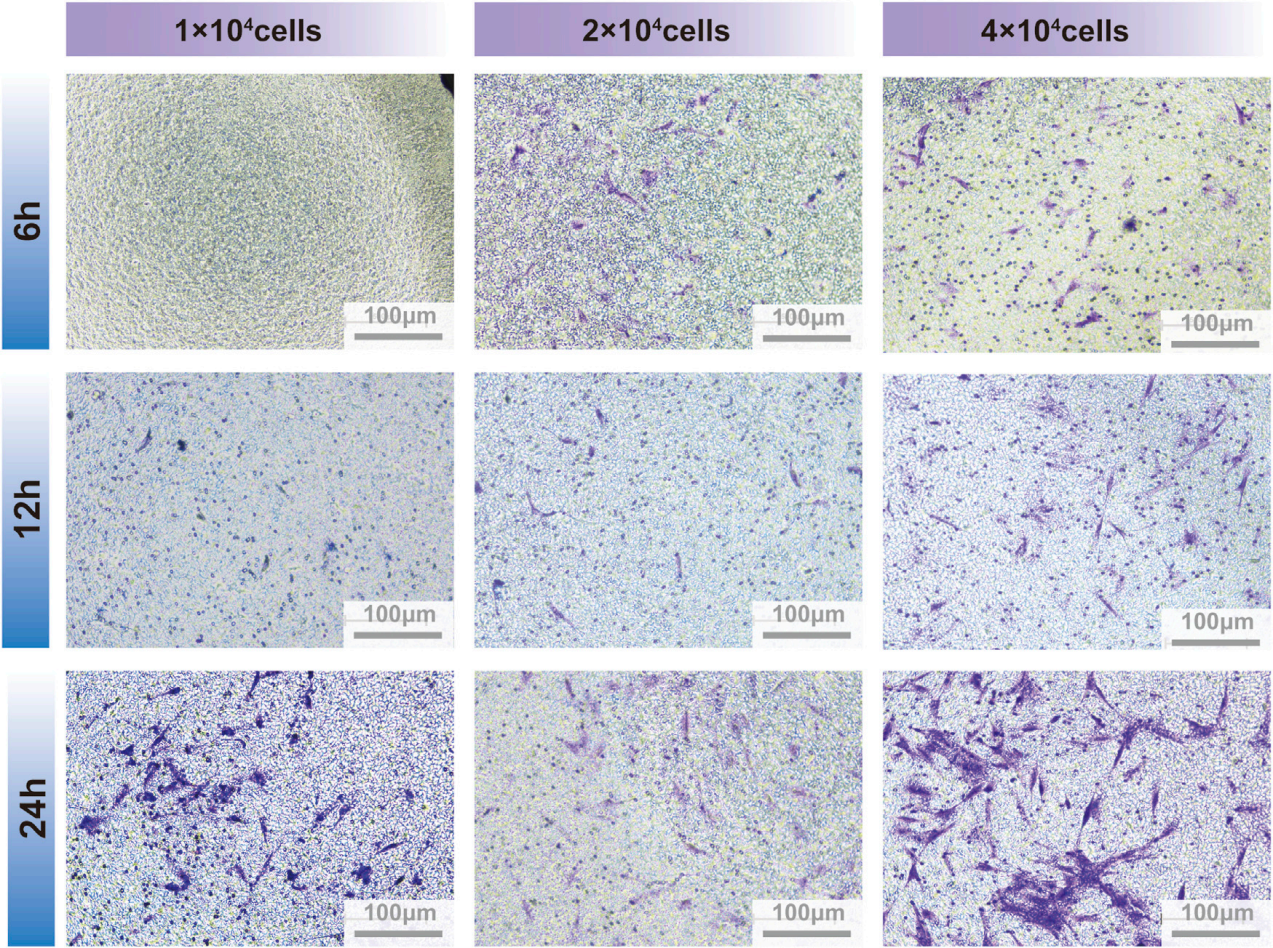
PDLSCs migration between transwell insert and lower chamber on different culture timing and cells quantity.

Fibroblasts move slowly *in vitro* and thus their cell migration were observed for the classic steps of locomotion (Trepat et al., 2012). However, mesenchymal cells possess the unique ability for collective migration, a crucial process for tissue remodeling *in vivo* and tissue engineering *in vitro.* During collective migration, external cues send signals that are sensed and responded to by the entire cell mass, thereby guiding the path of cell migration (SenGupta et al., 2021). The number of migrated PDLSCs treated with GQDs significantly increased at 24 h, while cells migrated sparsely at other times. At the time point of 24 h, the stained PDLSCs in 2×10^4^ cells well^-1^ were easily recognizable and appropriate in quantity for number counting, while the migratory cells of 4×10^4^ cells well^-1^ were numerous and agglomerated after staining with crystal violet, which apparently made counting difficult. Thus, the best time point and cell density were prepared by screening for further experimentation (Figure 4).

**FIGURE 5:**
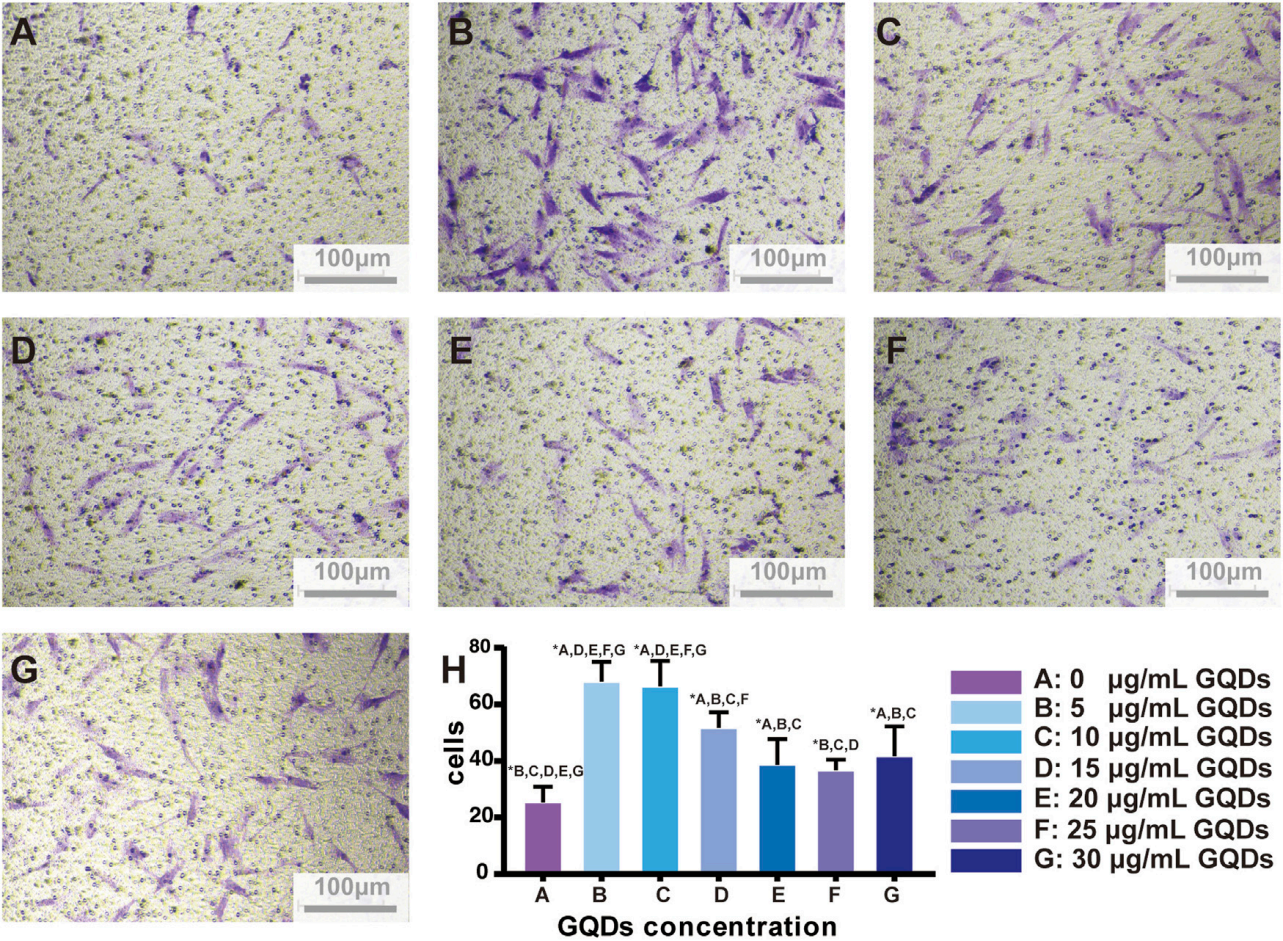
GQDs promotion effects on PDLSCs migration. **(A–G)** Migration-promoted effects of different concentrations of GQDs on PDLSCs. **(H)** Bar graph based on A-G. (A:0 μg mL^-1^, **(B)** 5 μg mL^-1^, **(C)** 10 μg mL^-1^, **(D)** 15 μg mL^-1^, **(E)** 20 μg mL^-1^, **(F)** 25 μg mL^-1^, **(G)** 30 μg mL^-1^, *p < 0.05).

PDLSCs migration speed was generally accelerated after culturing with GQDs. Within the range of 0–30 μg mL^-1^. Cell migration was highest at the concentration of 5 and 10 μg mL^-1^, and subtotal cells treated with GQDs significantly migrated faster compared with control groups (*p* < 0.05). (Figure 5). Based on above results and the effect of gradient concentrations of GQDs on PDLSCs viability, GQDs concentration of 5 μg mL^-1^ was used in further experiments.

Few studies have reported the effect of some GQDs composite biomaterials on cell migration. Cui *et al.* (2021) designed a smart dressing technology aimed at diabetic wound healing. It comprised of GQDs-containing luminescent porous silicon (GQDs @ PSi). The presence of GQDs on the PSi surface significantly enhanced the loading capacity of epidermal growth factor and insulin, which contributed to cell proliferation and migration and eventually promoted wound healing. Tung *et al.* (2020) designed a nanoscale radiosensitizer by grafting 2-deoxy-D-glucose onto GQDs to boost the efficacy of radiotherapy on osteosarcoma, and speculated that it might impair cancer cell invasion and migration by disrupting the F-actin cytoskeletal assembly. While these studies may not agree on the underlying mechanism of how GQDs affect cell migration owing to different assembly materials and experimental subjects, they still confirm that GQDs play a key role in cell physiology that is worthy of further exploration.

### 3.5 Differentiation of PDLSCs

**FIGURE 6:**
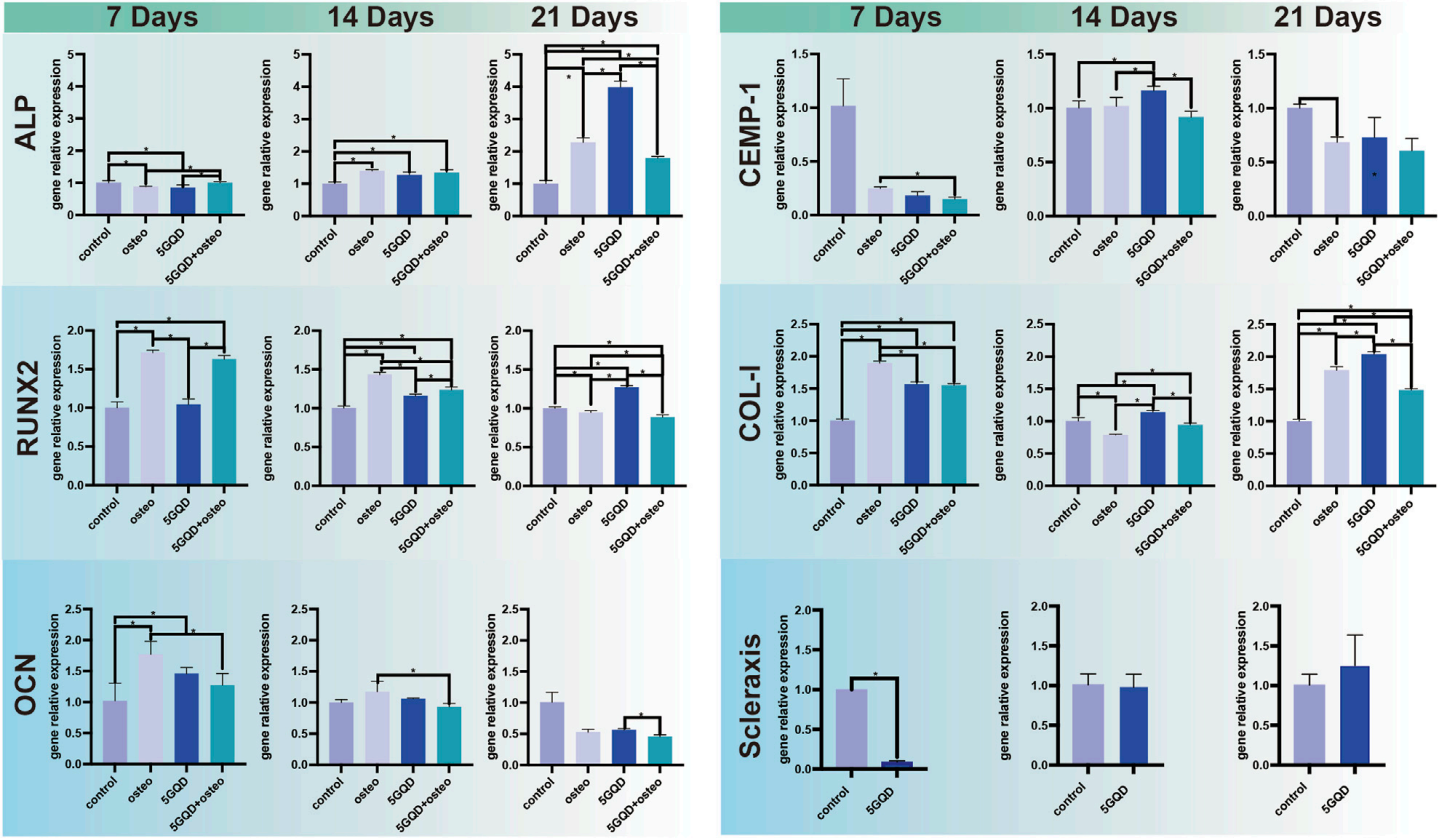
GQDs Effects on PDLSCs differentiation. ALP, RUNX2, OCN: osteogenic differentiation marker genes. CEMP-1: cementum marker gene. COL-I, Scleraxis: periodontal ligament relative fiber marker gene. *p < 0.05.

**FIGURE 7:**
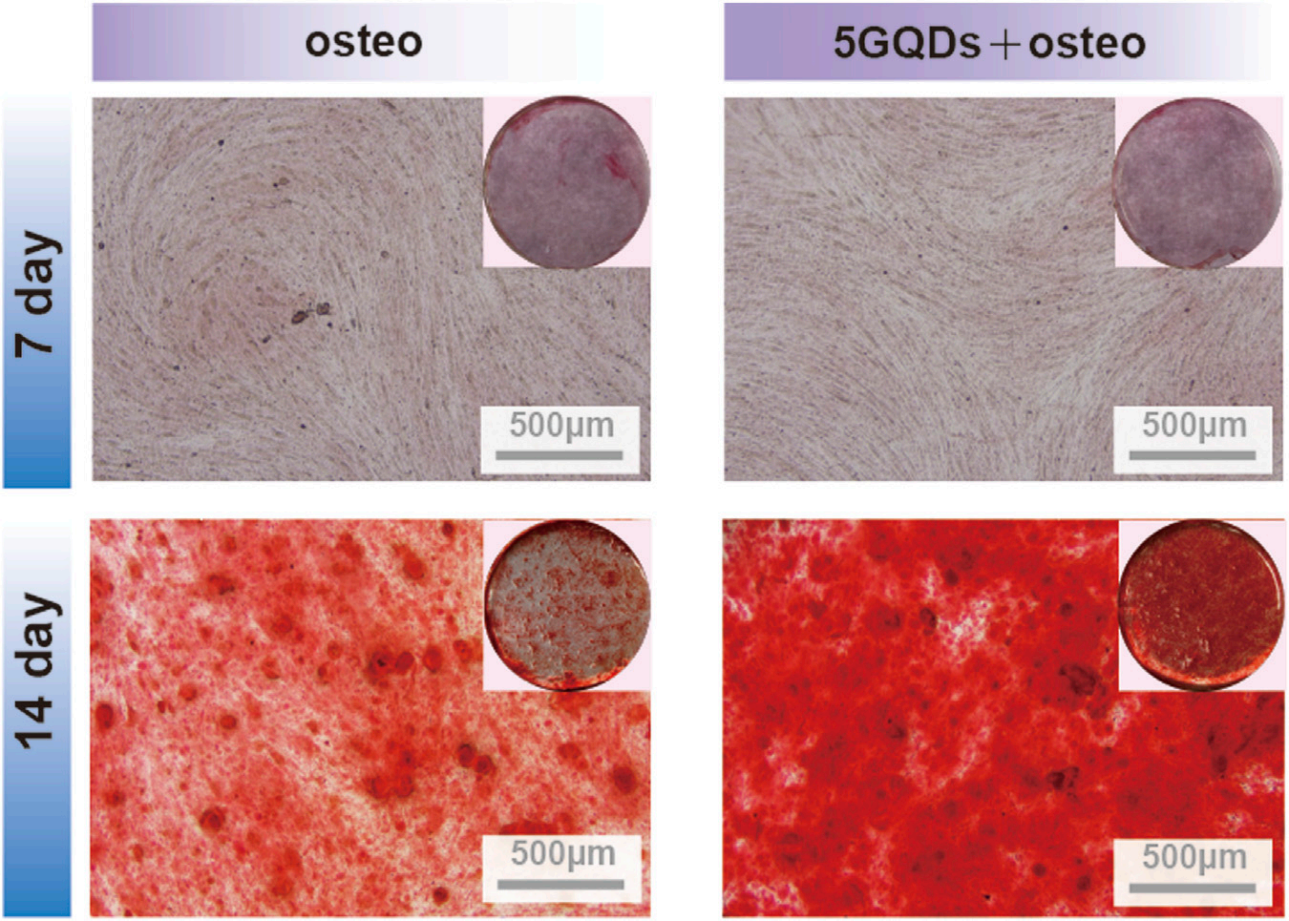
Osteogenic differentiation was detected by Alizarin Red S on day 7,14 in groups.

The expression of ALP, RUNX2, and OCN was distinctly higher in the experimental groups than in the control. Furthermore, the GQDs groups could induce the same osteogenic effect as osteogenic media could (Figure 6). ALP is closely related to the diagnosis and treatment of bone diseases and skeletal biology. As a marker of bone formation, ALP involved in physiological mineralization and shows rich activity in the early stage of mineralization; Runx2 is essential for osteoblast differentiation, and its expression reaches the maximal level in immature osteoblasts, which is a sign of osteoblast differentiation; OCN is a late marker of osteogenesis (Siller and Whyte, 2018; Carvalho et al., 2019). In this study, GQDs significantly promoted the RNA expression of ALP, and the relative expression level was much higher than that of the negative control group. The RUNX2 expression in experimental groups showed a different upregulating trend. The OCN expression showed the same upward trend in the early stage. The increasing trend in the Osteo and 5GQDs groups indicated that GQDs could spontaneously promote the osteogenic differentiation of PDLSCs even without the combination of osteogenic medium. To further confirm the effect of GQDs on the osteogenic differentiation of PDLSCs, ARS staining was conducted to verify whether there was mineralized nodule formation under material function. Mineralized nodule formation is shown in Figure 7. There was visibly higher calcium nodule formation in the 5GQDs-osteo groups than in the Osteo group at 14 days, which demonstrated that GQDs could induce osteogenic differentiation of PDLSCs.

The expression of several genes related to alveolar bone and cementum was validated by qRT-PCR. Obviously, the cementogenesis relative marker CEMP-1 expression was generally suppressed under osteogenic conditions, and GQDs did not work much on the expression promotion of CEMP-1. (Figure 6). CEMP-1 is a newly discovered product specifically expressed in the process of cementogenesis and has some unique roles in the commitment and the differentiation of cementoblasts. Overexpression of osteoblast related markers and mineralized nodule formation in ARS staining showed that PDLSCs cultured in osteogenic differentiation medium or GQDs-osteo medium, were differentiated towards bone cells. While CEMP-1 expression was reduced, which proved its expression did not correlate with osteoblastic differentiation, in accordance with the previous study (Komaki et al., 2012) research.

**FIGURE 8:**
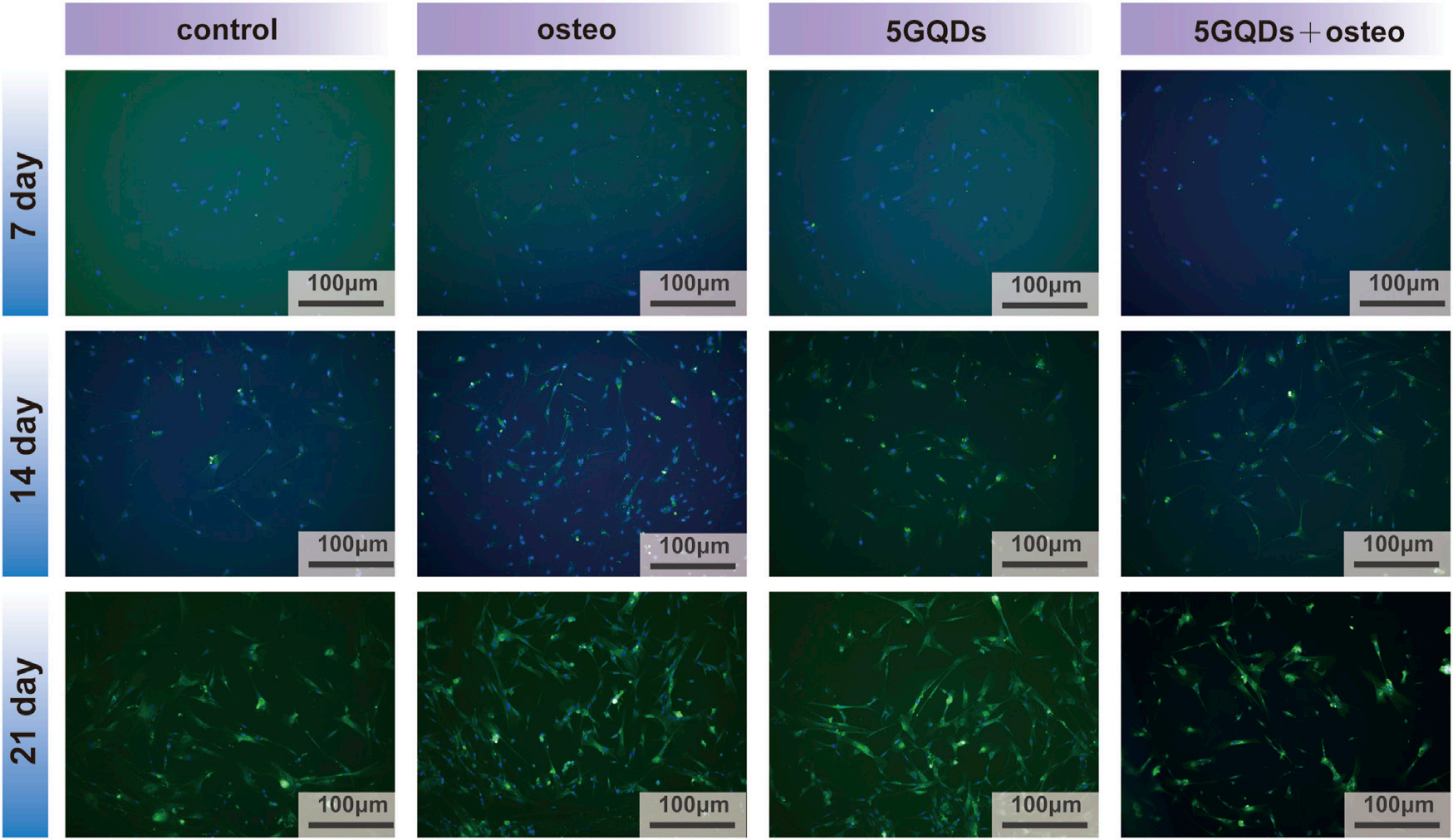
COL-I immunofluorescence staining of PDLSCs cultured by GQDs or/and osteogenic differentiation mediums on day 7, 14 and 21.

COL-I is not a typical bone specific marker because it presents in numerous unrelated cell types, however, it does work as a part in cell adhesion, proliferation and differentiation of the osteoblast phenotype and can be considered as an early indicator. And COL-I is also one of the main components of PDL tissue. COL-I genes were expressed at significantly higher levels in the experimental groups than in the control (Figure 6, *p* < 0.05). Interestingly, the combination of GQDs and osteogenic differentiation medium does not show a better effect result in the PCR process, this might relate to high expression in early stage due to combination of GQDs and osteogenic differentiation medium and it cannot maintain the trend in the late stage. Regarding the fluorescence results of COL-I, it was found in the present study that the fluorescence expression of the experimental groups was relatively strong at the three set time-points, indicating that both the osteogenic media and GQDs promoted COL-I generation in the cells (Figure 8). COL-I is the main component of PDL fibers and can represent the occurrence and development of PDL (Ullrich et al., 2019). It is also a marker gene and an essential fiber for the process of osteogenesis. In our study, COL-I expression in PDLSCs was upregulated by GQDs, which implied that GQDs could promote osteogenesis and fibroblast differentiation of PDLSCs. The expression of other marker—scleraxis in the GQDs group was significantly downregulated in the initial stage and not upregulated in the later stage (Figure 6). Recent research has found that scleraxis is a relatively specific molecular marker of tendons and ligaments that expressed at all stages of tendon development. The unique and dense collagen fibers of PDL are similar with those found in tendons, and both possess the ability to absorb mechanical forces. The scleraxis expression in PDLSCs was significantly higher than that in the other two mesenchymal stem cells—bone marrow stromal cells and dental pulp stem cells, thus scleraxis can be used as a specific marker gene for PDLSCs (Shi et al., 2005). In this experiment, scleraxis expression in the 5GQDs group was gradually upregulated in the middle and late stages, revealing that GQDs could promote fiber differentiation of PDLSCs.

Therefore, GQDs biological behavior towards PDLSCs have been investigated, and its advantages and disadvantages compared to other common bone tissue engineering biomaterials and graphene materials were summarized in Table 2 (Shi, et al., 2022; Fan, et al., 2023; Bharathi, et al., 2022; Yao, et al.,2023; Vahdat et al., 2019).

**TABLE 2:** Comparison of biomaterials on their advantages and disadvantages.

Material	Advantages	Disadvantages
GQDs (our work)	• Mechanical stability	• Further apply restricted by 0 dimension size
	• Biocompatibility	• Synthesis by chemical, electrochemical, or physical approaches
	• Cell migration	
	• Osteoinductivity	
	• Fibroblast differentiation	
	• Less toxicity and higher metabolic rate than other graphene materials	
Graphene oxide (GO), reduced graphene oxide (rGO)	• High mechanical strength	• Toxicity due to size
	• Biocompatibility	• Slow metabolic rate
	• Cell migration	
	• Osteoinductivity	
Hydroxyapatite (HA)	• Inertness	• Slow degradation rate
	• Hardness	• High elastic modulus
	• Abrasion resistance	• Higher fragility
	• Biocompatibility	
	• Osteoinductivity	
Collagen-Based Materials	• Excellent biocompatibility	• Poor mechanical properties
	• Cell adhesion	• Fast degradation
	• Osteoconductivity	• Lack of osteoinductivity
Chitosan	• Biocompatibility	• Decreased mechanical stability
	• Biodegradability	• Lack of osteoconductivity
	• Osteoinductivity	
	• Antimicrobial activity	

## 4 Conclusion

**FIGURE 9:**
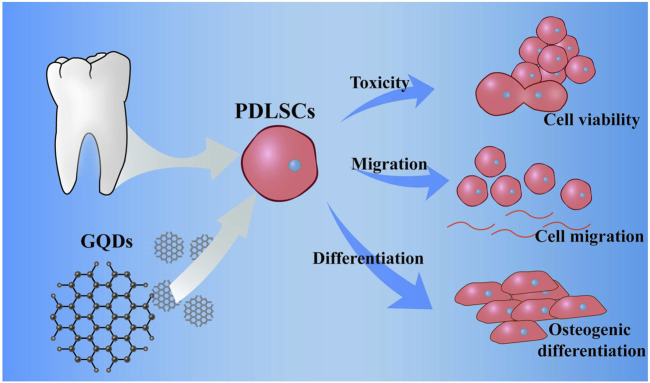
Scheme: culture process of PDLSCs, and promotion of the migration, differentiation of PDLSCs while coculture with GQDs.

In this study, we have uncovered a novel potential material GQDs for periodontitis treatment. GQDs exhibit robust mechanical strength and an impressive ability to expedite cell differentiation into bone cells. Our research confirms that GQDs have the remarkable capacity to stimulate osteogenesis and foster fibroblast differentiation within PDLSCs. GQDs not only possess low toxicity and excellent biocompatibility but also significantly enhance the migration of PDLSCs (Figure 9). Additionally, their chemical structure contributes to the mechanical stability of GQDs. What sets our study apart is the revelation of GQDs’ exceptional performance in inducing osteogenic fibroblast differentiation and promoting cell migration. This synergistic effect positions GQDs as a promising candidate for periodontitis therapy. Furthermore, these findings expand the scope of graphene materials in the realm of bone regeneration.

## Data Availability

The original contributions presented in the study are included in the article/supplementary material, further inquiries can be directed to the corresponding author.
